# Exploring Workers’ Experience in Public Administrations: Intergenerational Relations and Change as Difficulties and Potential

**DOI:** 10.3390/ejihpe16010014

**Published:** 2026-01-16

**Authors:** Cristina Curcio, Anna Rosa Donizzetti

**Affiliations:** Department of Humanities, University of Naples Federico II, Via Porta di Massa, 80133 Naples, Italy; cristina.curcio@unina.it

**Keywords:** ageism, gendered ageism, organisational well-being

## Abstract

Background: In a context of profound transformation within Public Administration, the growing generational diversity of the workforce poses critical challenges to organisational well-being. While ageism is a known risk, the intersectionality of age and gender—manifesting as gendered ageism—remains an under-explored area that can significantly undermine job satisfaction and employee health. Objective: This study aimed to explore the subjective work experience of public sector employees, specifically focusing on intergenerational relations and the impact of gendered ageism. Methods: A qualitative study was conducted involving 30 employees of the Italian Public Administration, recruited via purposive sampling. Data were collected through semi-structured interviews lasting approximately 38 min and analysed using a thematic analysis of elementary contexts via T-Lab software. Results: The analysis revealed four distinct thematic clusters positioned along two main factor axes (Individual/Organisation and Difficulties/Potential). The results show a dichotomy: while positive relationships with colleagues (Cluster 1) and the drive for change (Cluster 4) act as potential resources, the experience is marred by significant difficulties. These include organisational imbalances (Cluster 3) and, crucially, specific experiences of gendered ageism (Cluster 2), manifesting as stereotypes, pressure on women’s physical appearance, and exclusionary dynamics. Conclusions: The findings highlight that gendered ageism is a distinct stressor impacting workforce sustainability. Combating intersectional discrimination represents a strategic priority to safeguard well-being, retain skills, and build a healthy, resilient, and productive working environment.

## 1. Introduction

In today’s global landscape, characterised by increasing complexity and rapid technological, demographic and social progress, the study of change within Public Administrations (PA) is of crucial importance. Understanding the dynamics through which Public Administrations adapt and transform is essential to ensuring their effectiveness, their ability to respond to citizens’ needs and their long-term sustainability. The concept of change in the context of Public Administrations goes beyond simple operational changes, encompassing instead fundamental transformations in structures, processes, cultures and service delivery models (Rocha & Zavale, 2021).

In Italy, Public Administrations play a fundamental role, being a major employer and a key provider of essential services to citizens (Bulman, 2021). They operate in a dynamic and complex environment, subject to a wide range of internal and external pressures that require adaptation and change (Presidency of the Council of Ministers, 2021). External factors include political and regulatory changes (Capano & Natalini, 2020), public health crises such as the COVID-19 pandemic (ISTAT, 2021), rapid technological advances and the growing need for digital services (Agency for Digital Italy, 2024). Demographic changes are also significant external factors (ISTAT, 2024). The centrality of technology and digitalisation underscores the transformative impact of the digital age, which requires significant changes in service delivery models and work organisation to meet the growing demand for more transparent, accountable and responsive.

Internal factors driving change include the desire to improve performance, the need to evolve organisational culture, changes in leadership and the resulting priorities, the need to address performance gaps and respond to workforce needs (Rocha & Zavale, 2021). This wide range of factors, both internal and external, points to the dynamic and complex nature of the environment in which Public Administrations operate, which requires constant adaptation and change.

Among the most significant external factors are demographic changes. Italy, in particular, is undergoing significant demographic changes, characterised by a rapidly ageing population, declining birth rates and a growing proportion of older workers in the workforce (World Economics, 2025). Data show that in 2022, 37% of workers in Italy were between 50 and 64 years old (INAPP, 2023).

The ageing of the public sector workforce is not a phenomenon exclusive to Italy, but a trend observed in many OECD countries (OECD, 2021). Many developed nations are facing similar demographic changes in their Public Administrations. Although the general trend of an ageing public sector workforce is common, the specific age distribution and pace of change may vary between countries due to differences in birth rates, life expectancy, pension policies and labour market dynamics. Some countries may have a higher proportion of young workers in their public sector than Italy, while others may face even more pronounced ageing trends (EBSCO, 2023).

Further complicating the issue, alongside the phenomenon of the progressive ageing of the population and the workforce, is the fact that the contemporary world of work, including Public Administrations, in Italy and globally, is increasingly characterised by the coexistence of different generations, with the potential for up to five distinct generational groups to coexist (Fry, 2020).

This generational diversity presents both opportunities and challenges for organisational management and work culture, and makes it even more urgent to understand and effectively manage a multigenerational workforce in the public sector (International Monetary Fund, 2023). The loss of experienced workers due to retirement requires strategies for knowledge transfer and for attracting and retaining younger generations. The imminent retirement of a large segment of the workforce, particularly Baby Boomers, poses challenges for knowledge transfer and skills continuity. In addition, the rapid pace of technological change requires continuous adaptation and retraining of the workforce. Research suggests a tension between the need for innovation in Public Administration and potential resistance to change within established bureaucratic structures (Van der Voet, 2016).

The coexistence of multiple generations in the workplace undoubtedly offers a rich landscape of diverse experiences and skills, but it can also serve as fertile ground for the development of ageism (Butler, 1969). This issue is increasingly relevant in a work environment where Traditionalists, Baby Boomers, Generation X, Millennials and Gen Z coexist, each with their own characteristics and expectations (EBSCO, 2023). Examining the relationship between ageism and the chronological age of workers, it is evident that younger workers may have more severe ageist attitudes towards other age groups than older workers (Yi et al., 2022).

Within this discourse, the authors, in agreement with the literature, have chosen to refer to older workers as those aged 50 and above (McCarthy et al., 2014; Zacher & Rudolph, 2023), as the concept of older workers in the workplace develops based on workers’ progressive approach to retirement age. Ageism is therefore a phenomenon that potentially affects a significant portion of the workforce. Ageism in the workplace can take various forms. Research shows that older workers are considered inflexible, unwilling to adapt to technology, resistant to change and in a state of physical and mental decline, which is why they are often considered unsuitable for training and more expensive for the organisation (Johnson et al., 2013; Nelson, 2016).

For older workers, ageism can lead to decreased job satisfaction and motivation, hindering their commitment and productivity and limiting opportunities for advancement and professional growth. The negative consequences of ageism include decreased productivity, increased turnover, and a deterioration of the corporate climate (Yi et al., 2022). Older employees may also feel pressured to retire early due to discrimination and lack of opportunities (Donizzetti, 2015; Duncan & Loretto, 2004). Among older people, ageism is associated with poorer physical and mental health, increased social isolation, loneliness and stress, greater financial insecurity, decreased quality of life and premature death (World Health Organisation, 2021).

From an organisational perspective, ageism can have harmful consequences for Public Administrations. The early retirement of experienced older workers leads to a loss of valuable institutional knowledge and skills (International Monetary Fund, 2023). Ageism can stifle innovation and creativity by hindering collaboration and the exchange of ideas between different generations (Wang & Duan, 2024).

Ageism in the case of older women takes on a different and entirely unique form, giving rise to gendered ageism (Itzin & Phillipson, 1995). This refers to the specific discrimination and prejudice that arise from the intersection of age and gender. This distinction can be theoretically framed through the concept of “Double Jeopardy” (Beale, 1990; Clarke & Griffin, 2008), which posits that individuals belonging simultaneously to two marginalised categories face a double disadvantage. According to this perspective, the interaction between multiple forms of discrimination is not merely additive—i.e., the simple sum of ageism and sexism—but can be multiplicative. This creates a unique combinatory process where age and gender prejudices mutually reinforce each other, resulting in an even greater intensity of discrimination compared to that experienced by those with a single marginalised identity (Duncan & Loretto, 2004; Williams, 2014). In the workplace, this often manifests itself through unique challenges for older women, who experience the combined effects of ageism and sexism. This prejudice views feminine attributes associated with youth, such as being physically attractive, as factors that influence women’s work experience (Sigurðardóttir & Snorradóttir, 2020). Women face pressure to maintain a youthful appearance in order to be perceived as competent and relevant in the workplace. Unlike older men, whose grey hair may be seen as a sign of experience, wisdom and charm, older women may feel pressure to hide the signs of ageing (Cecil et al., 2023; McGann et al., 2016). In addition, women’s work performance is thought to decline earlier than men’s (Jyrkinen, 2014; McInnis & Medvedev, 2021). Women are seen as less competent because of their age and are less frequently asked to take on new responsibilities and tasks. This represents a major obstacle to career advancement. Older women are also more likely to be dismissed or encouraged to reduce their working hours and switch to part-time work (Sigurðardóttir & Snorradóttir, 2020).

The presence of gendered ageism in the workplace and its significant impact on job satisfaction (Monahan et al., 2023) and organisational climate (Tahmaseb-McConatha et al., 2023) could further complicate the process of change that the world of work, and Public Administrations in particular, have been facing in recent years. Despite the growing body of research on this topic, significant gaps remain. First, most studies in this field adopt quantitative approaches (Yi et al., 2022) that map the prevalence of discrimination but often fail to capture the nuance of the lived experience. Second, there is a lack of research specifically focusing on the intersectionality of age and gender within the Public Administration context, as the previous literature has often treated these dimensions separately (Krekula et al., 2018). Third, the construct of gendered ageism has not yet been operationalized, and there is currently no validated scale to measure it. Therefore, an in-depth exploratory understanding is required as a preliminary step before proceeding to any quantitative measurement. Consequently, adopting a qualitative approach is essential to address these limitations. This inquiry allows for an in-depth exploration of how structural organisational dynamics are interpreted and navigated by individuals on a daily basis, revealing the subtle, often invisible mechanisms of exclusion that surveys might miss.

Recognising these prejudices is a strategic necessity in order to focus on the potential of each worker, transforming the coexistence of different generations, combined with gender specificities, from a potential risk of conflict to a driver of innovation and public value.

Based on these premises, this study aims to explore, through a qualitative survey, the work experience of Public Administration employees. Specifically, the research aims to

1. Explore the quality of intergenerational relationships in multigenerational workplaces;
2. Investigate the perception and specific manifestations of gendered ageism compared to ageism;
3. Understand how these discriminatory dynamics intersect with perceived organisational well-being and the current process of change.

## 2. Materials and Methods

### 2.1. Procedures

Data collection took place between November and December 2023. Semi-structured interviews were used to collect data. The interviews were conducted in person, in a quiet setting to ensure privacy. The average duration of the interviews was approximately 38 min. The interview grid was constructed according to a thematic approach (Braun & Clarke, 2006). The main thematic areas were defined in line with the research objectives and based on the analysis of the relevant literature. The protocol was structured in two sections. The first included 9 items collecting socio-demographic and professional data; the second consisted of 22 open-ended questions formulated with the aim of stimulating the participant’s narrative, along with a series of follow-up questions to explore aspects of interest in more detail. A preliminary pilot phase was conducted with two participants (not included in the final sample) to ensure question clarity and refine the wording.

This approach ensured that all topics relevant to the research were covered, while maintaining the flexibility necessary to bring out the subjective experiences of the participants. The tool was designed to explore the perceptions and experiences of the subjects in relation to four main thematic areas: job satisfaction and dissatisfaction (e.g., ‘What satisfies you most about your job and what satisfies you least?’) perception of the organisational climate (e.g., ‘How would you describe the atmosphere in your office and more generally within your work environment?’) the quality of intergenerational relationships in the workplace (e.g., ‘How would you describe your relationships with younger/older colleagues?’) and experiences related to ageism (e.g., “How do you think your younger colleagues view your work abilities?”) and gendered ageism (e.g., “Do you think that for a female worker in the older age group, looking young or not can affect her work experience?”).

Participants were recruited through purposive sampling (Campbell et al., 2020). The selection was driven by the specific research objective to explore diverse perspectives within the Italian Public Administration context. Access to the participant pool was facilitated by a senior manager who provided official contact lists of employees, stratified by functional sector. To ensure a comprehensive representation of the public sector’s diverse working environments, the sample included employees from distinct functional areas, specifically: Legal Area, Administrative Area, Social Services and Citizen Services, Area Financial and Human Resources Area, Technical and Public Works Area, Urban Planning Area, Healthcare Area, and the Secretary General’s Office. Regarding the procedure to ensure balance, researchers selected participants from these sector-specific lists aiming for maximum heterogeneity. For each functional area, researchers specifically identified and contacted individuals to ensure the inclusion of both genders and different age groups (under and over 50), thereby preventing any single professional domain from being represented by a homogeneous demographic group. Participants were initially contacted by the researchers via their office telephone numbers to illustrate the project aims and verify inclusion criteria. According to the inclusion criteria, participants had to be adults working in Public Administration. Participation was voluntary. Participants were asked to respond honestly and were informed that they could withdraw from the study at any time. The research protocol was approved by the Ethics Committee for Psychological Research of the Department of Humanities at the University of Naples Federico II (prot. 22/2023 of 15 May 2023) and the University’s Privacy Office and was conducted in accordance with the ethical standards of the APA and the principles of the 1995 Declaration of Helsinki. Written consent to participate was obtained from each participant before the interview began. This consent included authorisation for audio recording, which was carried out to ensure accurate transcription of the content.

### 2.2. Participants

A total of 30 Public Administration employees took part in the study. Data collection continued until theoretical saturation was reached. This sample size meets the recommended standards for qualitative interview studies to identify meta-themes (Guest et al., 2006; Hennink & Kaiser, 2022). Participants were aged between 24 and 65 (M = 47.23; SD = 11.30), 14 were women and 16 were men. In terms of job category, 13 were managers and 17 were employees. Their length of service varied considerably, from a minimum of 1 year to a maximum of 40 years, highlighting the considerable heterogeneity of the group of participants (M = 11.97; SD = 13.04).

### 2.3. Analyses

The collected data were analysed following the methodological framework of Thematic Analysis (Braun & Clarke, 2006), according to which the analysis of qualitative data follows a process aimed at identifying, analysing and reporting patterns of meaning, i.e., themes, present in the textual corpus. In the first phase, all interviews were transcribed faithfully, forming a single corpus. The choice to use T-Lab, a package of linguistic, statistical and graphical tools for text analysis (Lancia, 2004), was driven by the need to reduce the potential for researcher confirmation bias and to validate the qualitative insights with a data-driven approach. Using this software, the corpus was first subjected to a process of disambiguation and automatic lemmatisation, which revealed that it consisted of 75,417 occurrences, 6626 forms and 4268 lemmas^1^.

Subsequently, the corpus underwent a Thematic Analysis of Elementary Contexts, a qualitative-quantitative analysis particularly suited to exploring the content of rich narrative and discursive corpora. This approach allows the patterns of meaning and recurring themes in the participants’ stories to emerge systematically. This analysis is carried out through a process that begins with the division of the text into Elementary Context Units (ECUs). ECUs represent the text segments into which the software divides the corpus for statistical processing, roughly corresponding to sentences or short paragraphs that convey a complete meaning. These are segments of text that are homogeneous in terms of lexical content and approximately the length of a sentence. These units are then classified and aggregated into thematic clusters based on the co-occurrence of specific keywords within them. The clusters are identified using an unsupervised hierarchical ascending method (specifically, the Bisecting K-Means algorithm). Each cluster consists of a specific vocabulary of keywords, whose statistical relevance within the cluster is measured by the chi-square test. In the final stage, researchers assign an interpretative label to each cluster, based on the vocabulary that characterises it. To ensure validity (triangulation), this labeling process and the interpretation of the factorial axes were performed independently by two researchers, who then discussed any discrepancies to reach a consensus. Furthermore, to visualise the relationships between these thematic clusters, the results are represented graphically using Correspondence Analysis. This graph positions the clusters as points on a Cartesian plane, whose structure is defined by two main axes. These axes are Factorial Axes extracted statistically from the software, representing the most important dimensions of meaning that organise the entire corpus. The horizontal axis (Factor 1) expresses the strongest semantic contrast, while the vertical axis (Factor 2) expresses the second most important contrast. The label assigned to each pole is derived from careful interpretation by the researchers, who analyse the groups of words at opposite ends of each axis to understand the logic of the contrast. In this way, the final graph not only shows the proximity or distance between themes, but also explains the fundamental relationships that define the participants’ experience, thus ensuring rigour and traceability in the qualitative interpretation of the data.

## 3. Results

### 3.1. Descriptive Analyses

The descriptive analysis classified 1654 elementary context units (ECUs) and divided them into four clusters, or macro-themes. With regard to the quantitative dimensions of the clusters: Cluster 1 consisted of 465 elementary context units, corresponding to 28.11% of the variance; Cluster 2 consisted of 479 elementary context units, corresponding to 28.96% of the variance; Cluster 3 consisted of 396 elementary context units, corresponding to 23.94% of the variance; finally, Cluster 4 consisted of 314 elementary context units, corresponding to 18.98% of the variance.

Table 1 shows the specific vocabularies for each cluster, sorted by T-Lab Plus according to the chi-square value.

**Figure 1:**
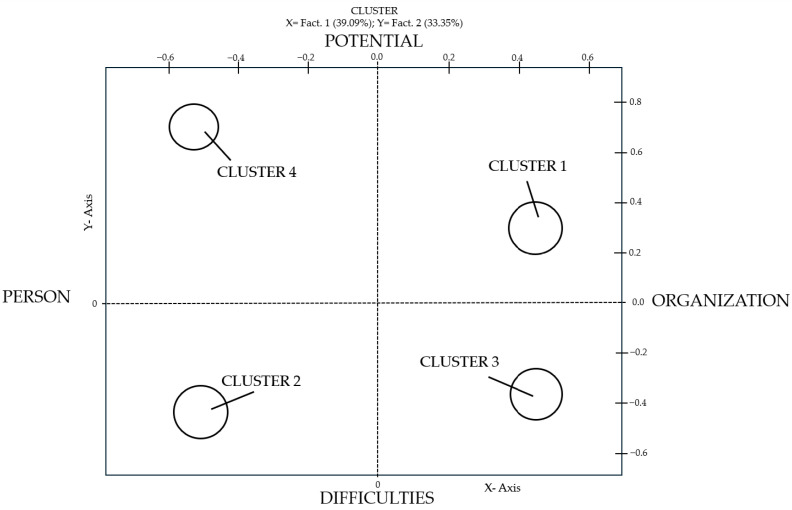
Factorial map showing the position of the clusters within the factor axes.

The relationships between these clusters are shown on a Cartesian plane in the Factorial Map (Figure 1). The Correspondence Analysis revealed a semantic space defined by two main factors, which together explain a significant portion of the total variance (72.44%).

Factor 1 (Horizontal Axis) explains 39.09% of the data inertia. It represents the focus of the discourse, distinguishing between the Person (Left pole) and the Organization (Right pole). The negative pole gathers themes related to the individual worker, their feelings, and personal issues, while the positive pole focuses on the structural dimensions and relational issues of the Public Administration.

Factor 2 (Vertical Axis) explains 33.35% of the data inertia. It represents the valence of the experience, contrasting Difficulties (Bottom pole) with Potential (Top pole). The negative pole highlights barriers, complaints, and critical issues, while the positive pole emphasizes resources, satisfaction, future perspectives, and positive change.

### 3.2. Cluster 1: Potential and Opportunities in Relationships with Colleagues

Cluster 1 is positioned in the top-right quadrant, at the intersection between Potential and Organization. It is characterised by words such as: work, colleague, relationship, excellent, change and contact.

This cluster highlights the positive elements that characterise relationships between employees. One element that emerges strongly from the analysis is the central role of interpersonal relationships as the foundation of organisational well-being. The interviewees’ words show that having a good relationship with colleagues is directly associated with enjoying the working environment. A young woman who has been working in the same Public Administration for some time said:

*«Personally, I believe I have a fairly peaceful relationship with all my colleagues, so I enjoy my working environment».*

Satisfaction with one’s job also seems to be influenced by the quality of the relationships established with colleagues. In this regard, a young male employee who recently joined the Public Administration said:

*«I also developed a rapport with the rest of my colleagues, which led me to appreciate the role and the sector I had been assigned to even more».*

This cohesion translates into an approach that sees results as the fruit of teamwork rather than individual effort. The emphasis shifts from the individual to the group, and success is perceived as the result of joint effort. An old man who recently joined the Public Administration said:

*«I would say that we try to do our job, at least in my sector, with my colleagues, in the best possible way».*

The same interviewee also shows us the pinnacle of this process, which manifests itself in prosocial behaviours that go beyond the formal boundaries of one’s own sector and duties. Solidarity and flexibility become the norm when the ultimate goal is the common good of the organisation. His words perfectly exemplify this mindset:

*«I have helped colleagues who may have had a heavier workload, helped colleagues from other sectors, even assembling chairs or drawers without any problems. If you do it, the important thing is to solve a problem».*

### 3.3. Cluster 2: Ageism and Gendered Ageism

Cluster 2 is positioned in the bottom-left quadrant, at the intersection between Difficulties and the Person. It is characterised by words such as: age, years, old woman and offend. This cluster brings together the critical issues perceived at the individual level and highlights the risk factors that workers associate with their own subjective experience. Among these, the issue of intergenerational comparison emerges as a significant source of tension.

Elements of prejudice towards older people emerge clearly from the words of a young manager:

*«Age has a big impact on jokes; it’s a very relevant issue. For example, men are said to lose their minds when they reach a certain age».*

Conversely, older workers recognise that they suffer from such prejudice, reacting with a strategy of defensive distancing, as is evident from the words of an older interviewee:

*«They tease me by calling me “grandfather” or “uncle” … I say, “Listen, mate, you’re thirty or forty years old, but I don’t compare myself to you».*

The interviewees’ words also reveal a conflictual dynamic where older people are seen as stingy holders of knowledge, while young people are seen as active perpetrators of prejudice. A young woman effectively sums up this conflictual dynamic:

*«Older people tend to keep their knowledge to themselves, not to divulge it, not to pass it on, while young people tend to say: “You’re getting on in years, you’re ready for retirement, you’re slow, you’re not as efficient as I am, so I’m better than you».*

The issue becomes more complex and multifaceted when age intersects with gender, giving rise to the phenomenon of gendered ageism. Stereotypes such as: ‘you shouldn’t ask a woman her age’ are interpreted by some male interviewees not as a vulnerability for women but as a strategic tool. As one man stated:

*«Maybe she doesn’t care about her age, but since there is this idea that ‘you shouldn’t ask a woman her age’, then she perceives it as a lack of gallantry, perhaps».*

This interpretation culminates in a mechanism in which the responsibility for the discomfort is entirely attributed to the women themselves. The same interviewee stated:

*«We need to make it clear that a woman should not be offended… she needs to accept her age».*

Another dimension of gendered ageism concerns social pressure on physical appearance, which weighs heavily on older female workers. An older man observed:

*«The only thing that destabilises them is not the workplace, but their age […] when they reach forty, they already feel old. Because they are confronted with the issue of appearance. Today, it’s all about appearance, beauty».*

Direct testimonies of older female workers confirm this pressure. A first defence mechanism is psychological distancing from the stigmatised category, as emerges from the words of an older interviewee:

*«Regardless of that, I don’t feel like an old woman».*

This distancing is not confined to identity, but translates into concrete behaviours aimed at masking the signs of ageing, as the same woman describes:

*«Despite my white hair, which I cover up every 20 days so that no one can see it».*

Another issue highlighted by this old woman concerns the discrimination she experiences from her male colleagues, who seem to prefer younger female colleagues:

*«The older male colleague has more fun, showing off with the thirty-year-old colleague rather than the fifty-year-old one, yes, that’s how it is».*

The emotional weight of these dynamics is heavy. Another interviewee describes the deep distress caused by age-related insults, to which the woman tries to respond by verbally expressing her discomfort:

*«It’s just a matter of feeling bad because I received that insult. But all I can say is: “I was hurt that you said that to me, because it’s true, yes, I feel that I am of a certain age, I don’t disagree with you, but you know that’s the way it is, so why are you criticising me?”».*

Finally, a feeling of inadequacy emerges in relation to atypical career paths, such as being hired at an advanced age. Another woman says she feels out of place because she was hired at the age of 52:

*«I feel out of place in my current job […] but in this case it is my age that makes me feel out of place. It is precisely because I was hired at the age of 52 on a permanent basis in the Public Administration».*

### 3.4. Cluster 3: Challenges in Managing Working Relationships

Cluster 3 is positioned in the bottom-right quadrant, at the intersection between Difficulties and Organization. It is characterised by words such as: manage, deal with, difficult and organise. This cluster explores the critical issues that emerge from the interpersonal dynamics within the organisation structure. Among the elements that seem to generate discontent are the imbalances that sometimes exist between different sectors and professional figures. A female manager noted:

*«What I like least, from a general point of view, is the organisation of the institution, because in my opinion there are some imbalances between the various sectors and roles».*

This structural weakness has a direct impact on the day-to-day management of relationships, making it a difficult and delicate task. A young manager highlights the presence of a fragile balance, which requires extra attention:

*«It is also difficult to manage relationships with other employees. Having a solid structure in which to operate is certainly important […] but you also need to have the sensitivity to know how to manage the balance».*

To make the management of interpersonal relationships even more complex, there are a number of stereotypes regarding the difficulties that different social categories may have in the workplace. The same young manager, for example, reflects on a supposed lack of relational experience on the part of younger people:

*«Perhaps simply with a more mature management of relationships, which a young person certainly cannot have».*

But he also reflects on the greater ability of young people, compared to older people, to work in a team:

*«Working in teams is always within the capabilities of young people».*

The challenges of teamwork are also filtered through a gender lens, giving rise to stereotypes about women’s interpersonal skills. Another young manager articulates a dichotomous view of men’s and women’s abilities:

*«Perhaps women have greater precision […] but perhaps men have greater teamwork skills than women».*

Finally, the point of view of older workers regarding the integration of new hires emerges. A senior female worker recognises the academic skills of young people, but identifies barriers that prevent the establishment of positive interpersonal relationships:

*«Even though the young people […] have their own, ‘fresher’ educational background, some of them are full of themselves. So let’s say that relations are limited to good office neighbours».*

However, his reflection ends on a hopeful note, suggesting that initial friction can be overcome with time, leaving open the possibility of future integration:

*«Then, over time, relationships can improve, that’s for sure».*

### 3.5. Cluster 4: Change in Public Administration and Work-Life Balance

Cluster 4 is positioned in the top-left quadrant, at the intersection between the Person and Potential. It is characterised by words such as: today, career, young person, arriving, progression and children. This cluster highlights how the transformations taking place in Public Administration are perceived and experienced subjectively. A central theme is the perception of an epochal change that is sweeping through the public sector. As one senior employee states:

*«Because today we can see that the world has changed».*

This change is identified by a young manager with the advent of so-called ‘administration 2.0’, driven primarily by technological innovation, whose evolution is seen as unstoppable:

*«In recent years, there has been a change in Public Administration, administration 2.0 […]. We’ve reached the age of the computer, but today it is the computer, tomorrow it will be artificial intelligence».*

In this scenario, young people are seen as the main repositories of new digital skills, a generational gap well described by an older worker through a comparison with his own youth:

*«Today, young people, children, are already growing up in a certain way with regard to computers, smartphones… When I was a boy, our games were table football and pinball, but I also notice this with my children who play on the PlayStation».*

In addition to technology, another driver of change is identified in the recruitment of new recruits, whose arrival is seen as a positive factor. An older manager said in this regard:

*«The most recent staff recruitment took place in 2019. Many talented young people have joined us».*

Intergenerational contact in this context of renewal can become a valuable resource, capable of generating individual well-being, as one senior employee testifies:

*«Even though I am 65, I feel like a youngster among the young people».*

However, within these dynamics of change, a specific critical issue emerges regarding work-life balance, particularly for women. A senior manager with long work experience describes how taking on a managerial position can become unsustainable, leading to resignation in order to safeguard one’s well-being and health, even in the face of economic advantage:

*«The worst period of my working life was precisely when I had a managerial position […] for a woman who wants to balance her career and family with a managerial position […] because if I have to sacrifice my health to be a manager, honestly, no […]. I want the salary I earn today and to be healthy and peaceful, to come home peaceful».*

This search for a new balance has had a positive outcome in the experience of a young woman who, through a change of job, has rediscovered her well-being by prioritising time for her family:

*«Now I can be with my son. My son is 7 years old today, so I’m home at Christmas, I’m home at Easter, I’m home on Saturdays and Sundays, whereas before I was forced to go to work anyway, and it was the happiest choice of my life».*

## 4. Discussion

This study aimed to explore the work experience of Public Administration employees, with a focus on intergenerational relationships, ageism, gendered ageism and their intersections with satisfaction and organisational climate. The qualitative analysis revealed a complex reality, allowing for the reconstruction of the workers’ experience. The results highlight a clear dichotomy between the individual and organizational dimensions defined by the factorial axes. On the organizational side (Right Axis), a dual dynamic emerges: on the one hand, interpersonal relationships act as a protective factor and a source of potential (Cluster 1), while on the other, structural and managerial inefficiencies are perceived as the primary barrier to well-being (Cluster 3). On the individual side (Left Axis), the experience is similarly polarized. Workers perceive the potential for personal growth and better work-life balance driven by modernization (Cluster 4), but simultaneously suffer from the subjective burden of ageism and gendered ageism (Cluster 2). In summary, this general model suggests that while the Public Administration offers significant relational and evolutionary resources (Top Axis: Potential), it remains burdened by cultural stereotypes and structural rigidities (Bottom Axis: Difficulties) that negatively impact the workforce, particularly older women.

Moving beyond description, these findings offer significant insights. First, the ‘Potential’ axis aligns with the principles of Positive Organizational Psychology (Luthans, 2002; Donaldson & Ko, 2010). Specifically, the finding in Cluster 1 that good relationships are central to satisfaction validates the Job Demands-Resources (JD-R) model (Bakker & Demerouti, 2007) within the Italian public context. Similarly, in Cluster 4, the advent of ‘administration 2.0’, driven by technology (Agency for Digital Italy, 2024) and the inclusion of new generations (International Monetary Fund, 2023), is perceived not just as a change, but as an opportunity for professional enrichment. Our data suggest that in a rigid bureaucratic environment, these two dimensions act synergistically as crucial compensatory job resources. Together, they buffer the negative impact of organisational demands, generating the resilience necessary to navigate the complex transition of the PA. This combination of social support and individual drive for change represents a strategic lever for improving performance and organisational culture (Rocha & Zavale, 2021; Presidency of the Council of Ministers, 2021).

Conversely, the ‘Difficulties’ axis expands upon Generational Diversity Theories, particularly the Intergenerational Tension Model (North & Fiske, 2015). The conflictual narratives observed in Cluster 2 are not isolated incidents but reflect the theoretical struggle over succession and resource consumption. In a context of high job security and low turnover (Di Mascio & Natalini, 2015; OECD, 2021), younger employees perceive older generations not as mentors but as barriers to resource consumption and career advancement. This structural blockage triggers the enactment of prescriptive stereotypes (e.g., ‘older workers should step aside’) to delegitimize the older out-group, creating an ‘Us vs. Them’ dynamic consistent with Social Identity Theory (Tajfel & Turner, 1979). Furthermore, the emergence of gendered ageism within this cluster challenges traditional Organizational Climate models (Schneider et al., 2013). Our data show that the organizational climate is not experienced uniformly; rather, exclusion operates through intersectional lenses (Cecil et al., 2023). For women, the pressure to ‘mask’ their age to remain professionally visible demonstrates that the ‘difficulty’ is not just about competence, but about the aesthetic labor required to navigate a system that penalizes the intersection of female gender and aging. Crucially, Cluster 3 demonstrates how these individual prejudices solidify into structural barriers. The perception of ‘imbalances between sectors’ and the difficulty in ‘managing balances’ are not merely logistical issues; rather, they represent the institutionalisation of the ‘Us vs. Them’ dynamic described by Social Identity Theory (Tajfel & Turner, 1979). Instead of integrating, the generational groups tend to segregate, protecting their respective boundaries. The stereotypes emerging here regarding interpersonal skills—such as the belief that young people lack relational depth or that ‘men have greater teamwork skills’—function as gatekeeping mechanisms. These biases justify the exclusion of certain groups from key collaborative roles, preventing the formation of diverse teams. Consequently, this lack of genuine integration reduces intergenerational relations to the status of ‘good office neighbours’, a form of passive coexistence that blocks the generative interaction essential for innovation (Wang & Duan, 2024).

This study contributes to filling a gap in the literature by addressing two main limitations in current research. First, it answers the specific call for a more intersectional approach to age discrimination (Krekula, 2007; Riach, 2009). While prior research has often treated age and gender biases as separate phenomena and measured them through broad quantitative scales, our findings on gendered ageism illuminate the lived, qualitative mechanisms of exclusion—such as ‘masking’ and social invisibility. This adds a crucial layer of depth to the understanding of how organizational culture implicitly perpetuates discrimination against older women. Second, the study provides unique insights into the dynamics of ageism within highly bureaucratic and low-mobility contexts. While international studies often focus on dynamic private-sector environments (Posthuma & Campion, 2009), our results highlight the specificities of the Italian Public Administration. Here, the “forced coexistence” resulting from high job security and a traditionally rigid turnover system (Di Mascio & Natalini, 2015; OECD, 2021) creates a distinct environment. Unlike in flexible labour markets, intergenerational tensions here risk turning into organisational paralysis rather than exit turnover. This suggests that in such contexts, mere physical proximity between generations is insufficient to foster integration without active management strategies.

### 4.1. Practical Implications

The Public Administration is an organisation in transition, rich in human and relational potential but at the same time afflicted by prejudices and discrimination that undermine its cohesion and effectiveness. The challenge for management and future policy will be to harness the potential (good relations, the drive for change) while actively addressing the difficulties (ageism, gendered ageism and management issues), transforming generational diversity from a risk into a real driver of public value. The complex landscape emerging from the results necessitates a multi-layered intervention strategy for public management.

Firstly, regarding intergenerational policies, the findings suggest that unstructured contact is insufficient to abate the conflict observed. Therefore, institutions should implement structured mutual mentoring programmes. Unlike traditional mentoring, this reciprocal approach—where seniors share institutional memory and juniors transfer digital competencies—helps to break down the status hierarchy that fuels conflict, operationalising the contact hypothesis in a constructive manner (Allport, 1954; Pettigrew & Tropp, 2006).

Secondly, the prevalence of stereotypes indicates an urgent need for managerial training focused on age management (Burnes et al., 2019). Leaders must be equipped to recognise and dismantle unconscious bias in daily interactions. This should be supported by the introduction of regular internal audits specifically designed to detect ageist language and practices, making ageism as visible and unacceptable as other forms of discrimination.

Thirdly, to address the “glass ceiling” (Morrison et al., 1987) identified in Cluster 4, a revision of promotion practices is required. The frustration expressed regarding career progression suggests that current evaluation and progression mechanisms may be permeable to bias. Ensuring transparency and objectivity in these processes is crucial to prevent gendered ageism from becoming a structural barrier to career advancement.

### 4.2. Limitations and Future Research

This study is subject to specific limitations that must be considered. The primary limitation concerns the sampling methodology; the use of purposive sampling, while effective for reaching specific targets, relies on the researchers’ discretionary judgment. This non-probabilistic approach inherently prevents statistical generalisation and may have introduced selection bias, potentially privileging participants who were more accessible or perceived as ‘key informants’, while overlooking less visible perspectives. Furthermore, the study is deeply rooted in the cultural specificity of the Italian public sector. The unique combination of high job security and an aging workforce creates distinct dynamics that may not be directly generalisable to private sectors or different national contexts with more flexible labour markets. Finally, the cross-sectional nature of the research captures a snapshot during a specific transition period and cannot determine causality. Despite these limitations, the study opens avenues for future research. It would be useful to conduct large-scale quantitative studies to measure the prevalence of the identified phenomena of ageism and gendered ageism and their correlation with performance and well-being indicators. To this end, future research should prioritize the development of a validated scale specifically for gendered ageism, which is essential to fully capture the intersectional nature of the phenomenon often missed by generic measures. The rich qualitative evidence gathered in this study offers an empirical groundwork for the structuring and wording of such items. Longitudinal studies could monitor the evolution of these dynamics over time, especially in relation to the implementation of new technologies and the National Recovery and Resilience Plan (Minister for Public Administration, 2024). Finally, intervention research could design and evaluate the effectiveness of training programmes and organisational interventions aimed at countering prejudice and promoting a truly inclusive and intergenerational work culture.

## 5. Conclusions

This study explores work experiences within the Italian Public Administration at a time of profound demographic and technological change. The results reveal a complex map of the working environment, dominated by a fundamental tension between the potential offered by human relations and change, and the difficulties generated by deep-rooted prejudices and management challenges. In particular, this research highlights the pervasiveness of ageism and, in an even more insidious form, gendered ageism.

Crucially, the explicit novelty of this study compared to the prior literature lies in its qualitative operationalization of gendered ageism within a low-turnover bureaucratic context. While previous research has predominantly relied on quantitative scales to measure ageism and sexism as separate constructs, this work empirically demonstrates how these prejudices interact to create unique mechanisms of exclusion—such as ‘masking’ and social invisibility. Furthermore, unlike studies focused on the private sector, our findings reveal a specific dynamic of ‘structural paralysis’ typical of the ‘forced coexistence’ in the Public Administration, offering a new theoretical lens for understanding organizational immobility.

The main conclusion of this work is that the success of the modernisation of the Public Administration (Presidency of the Council of Ministers, 2021) cannot be separated from a targeted investment in human and relational capital. Actively combating all forms of age and gender discrimination is not only an ethical imperative (World Health Organisation, 2021), but also a strategic necessity in order to retain knowledge and skills (International Monetary Fund, 2023), promote a positive organisational climate (Tahmaseb-McConatha et al., 2023) and unleash the innovative potential that arises from collaboration between different generations (Wang & Duan, 2024).

Based on the evidence collected, this study proposes clear policy recommendations for public management. To move beyond the current tensions, the Public Administration must implement targeted strategies. First, structured intergenerational training programs are necessary to facilitate knowledge transfer. Second, organisations should conduct regular internal audits on ageism and gendered ageism to monitor the organisational climate. Third, a rigorous review of promotion procedures is required to ensure they are free from gendered and ageist discriminations.

## Figures and Tables

**Table 1:** Vocabulary of each cluster and Chi Square.

CLUSTER 1		CLUSTER 2		CLUSTER 3		CLUSTER 4	
Lemma	Chi Square	Lemma	Chi Square	Lemma	Chi Square	Lemma	Chi Square
work	189.941	age	177.928	certainly	114.875	today	122.726
colleague	177.056	years	84.491	to succeed	98.040	to take	105.332
sector	96.500	fact	70.830	to manage	57.885	career	69.546
Public Administration	65.528	to feel	70.263	determined	56.131	to specify	65.809
internal	60.345	you	69.343	to determine	54.929	memory	54.283
relationship	41.518	your	54.993	to create	48.677	administration	47.696
excellent	35.655	to understand	52.463	to talk	41.610	public examination	43.602
to carry out	34.843	fifty	49.885	ability	39.942	boy	41.835
to find	33.873	Older woman	44.197	to face	31.205	to arrive	39.768
employees	32.585	oh well	41.987	important	30.820	own	37.220
financial	30.415	old	39.576	to need	30.006	to think	35.253
office	24.682	respect	38.708	difficult	27.922	to enter	34.917
change	24.462	woman	32.134	historical	26.460	month	33.851
new	21.207	to offend	29.568	to show	25.563	progression	32.891
responsible	21.045	thirty	28.737	to inform	24.116	child	28.100
our	20.933	issue	27.301	to notice	19.347	children	27.616
citizen	20.757	man	24.798	memory	19.170	way	27.616
climate	18.324	she	23.778	competence	18.677	to play	26.960
contact	16.302	hair	23.551	to pass	18.131	to hope	26.142
environment	15.508	understood	23.258	to organise	17.860	precise	23.152

## Data Availability

The data presented in this study are available on request from the corresponding author due to privacy restrictions.

## Note

1 *Occurrence* refers to the total number of words that make up the text. *Form* refers to each individual word, distinguished from others solely on the basis of its graphic structure, which may occur any number of times in a corpus or text. The *lemma* is the element to which a set of forms are referred, which are distinguished from each other because they are the result of the inflection of the same verb, noun or adjective.
